# A Systematic Approach to Building High-Impact Aging Datasets for Researchers

**DOI:** 10.1093/geroni/igaf122.3085

**Published:** 2025-12-31

**Authors:** Riya Goyal, Yater Henry, Tasnim Raisa, Jessica Fleck, Duo Wei

**Affiliations:** Stockton University, Galloway, New Jersey, United States; Stockton University, Galloway, New Jersey, United States; Stockton University, Galloway, New Jersey, United States; Stockton University, Galloway, New Jersey, United States; Stockton University, Galloway, New Jersey, United States

## Abstract

Access to relevant and high-quality secondary datasets is essential for advancing research on the older population across diverse domains such as mental health, health services, and life satisfaction. This study presents a systematic approach to identifying and curating a searchable database of the most significant secondary datasets in these fields. Our method begins with domain experts defining key content domains and their respective subdomains. Using Claude AI, we conduct targeted searches by combining subdomains with data analytics keywords and the term “older adults” to retrieve relevant datasets. To assess the importance of each dataset, we develop a Python algorithm that analyzes the frequency of dataset occurrences across multiple search combinations. For instance, by leveraging Claude AI to generate 100 datasets through a query of “older adults,” “Alzheimer’s,” and “regression,” we subsequently matched these datasets against PubMed’s repository, revealing 7,561 relevant publications, with 7,030 specifically addressing aging research. This iterative process allows us to identify the most commonly used and impactful datasets for researchers in aging studies. The resulting database will provide a structured, accessible, and efficient resource for scholars and practitioners seeking high-value secondary data to support evidence-based research on aging-related topics.

